# Developing a tool for the measurement of social exclusion in healthcare settings

**DOI:** 10.1186/s12939-022-01636-1

**Published:** 2022-03-15

**Authors:** Patrick O’Donnell, Ailish Hannigan, Nuha Ibrahim, Diarmuid O’Donovan, Khalifa Elmusharaf

**Affiliations:** 1 School of Medicine, University of Limerick, Limerick, Ireland; 2 Health Research Institute, University of Limerick, Limerick, Ireland; 3 Centre for Public Health, School of Medicine, Dentistry and Biomedical Sciences, Queens University, Belfast, Northern Ireland

**Keywords:** Social exclusion, Health, Intersectionality, Scale development, Tool evaluation

## Abstract

**Background:**

Social exclusion is a complex concept that is recognised as a key determinant of health. Many measurement tools developed looked at people from single excluded groups in isolation. We know from experience and literature that exclusion is often intersectional and multi-layered. Therefore, the aim of this research was to develop a social exclusion measurement tool for use in healthcare settings with individuals from any excluded group that would include questions to investigate socioeconomic elements and subjective experiences in their lives.

**Methods:**

Inductive and deductive methods were used to develop the tool. Early drafts were tested with experts (both academic and experts by experience) and modified in line with feedback received. The tool was then piloted with people in the community, and this allowed us to assess the internal consistency and validity of the tool. Exploratory factor analysis was carried out as part of this evaluation.

**Results:**

The measurement tool was initially evaluated by 17 academic and ‘real world’ experts. It was then piloted with seven experts by experience, two gatekeepers and two participants who were presumed not to be excluded, resulting in the development of the final tool. This was then tested with 276 participants (127 presumed excluded, 149 presumed not excluded). The socioeconomic characteristics of these participants were documented, and exploratory factor analysis was carried out on data relating to subjective items. A four-factor structure emerged comprising 22 items. Internal consistency of the factors was high, and their ability to discriminate between the two groups was notable.

**Conclusions:**

A tool for measuring the social exclusion of individuals has been developed by engaging with people from a variety of excluded groups. Socioeconomic indicators were combined with subjective items. The input of experts by experience, academics and others was sought to enhance the tool. The tool was applied to two distinct samples, showing obvious differences both in the socioeconomic items, and the items included in the factor analysis. The potential use of this tool could have positive implications for people who are excluded.

**Supplementary Information:**

The online version contains supplementary material available at 10.1186/s12939-022-01636-1.

## Background

Social exclusion (SE) is a complex concept that has been debated over many years and across many academic disciplines. SE is thought to have been first clearly described by René Lenoir in his writings about “les exclus” in France; these were people who had fallen outside of the reach of national welfare programmes designed to support all French citizens [1]. Since then, international organisations and representative bodies such as the World Bank, the European Commission, the World Health Organization (WHO) and others have explored the concept and sought to operationalise it [2–4]. There have been many definitions of SE published: essentially they describe disadvantage faced by particular groups and individuals who are felt to be removed from mainstream society, and who cannot fully participate in what that society considers ‘normal’ life [5]. SE has close links with the concept of poverty and material deprivation, highlighted by Atkinson [6] who wrote that any “analysis of social exclusion can broaden the discussion of wellbeing by considering dimensions beyond income poverty … Being poor can lead to exclusion, but exclusion is more than just being poor, it is about participation”. The use of the term SE in policy discourse has been met with scepticism by some commentators and scholars. They are critical of any move away from focusing on low levels of income as the predominant cause of disadvantage and marginalisation; worrying that this approach may indicate that responsibility for being socially excluded rests with individuals themselves, shifting the focus from those with power in societies [7–9]. The concept of intersectionality is also relevant to SE as it acknowledges that many individuals are subject to multiple forms of disadvantage and marginalisation that combine to result in increased discrimination and adverse health outcomes [10]. On discussing the intersectionality perspective, Havinsky [11] explained that inequities are “never the result of single, distinct factors. Rather, they are the outcome of intersections of different social locations, power relations and experiences.”

It is now accepted that SE is inextricably linked to the health and wellbeing of people affected by it [9, 12–14]. This was highlighted in 2008 by the publication of the Social Exclusion Knowledge Network (SEKN) report for the WHO Commission on the Social Determinants of Health [4]. The SEKN group described exclusion as having many dimensions, that it was a dynamic process, and that it could occur at many levels – from the individual to the global. It also recognised that social inclusion (SI) and social exclusion were on a continuum, greatly influenced by access to resources, the ability to realise one’s rights and the capabilities of people to put those rights and resources to use. Groups that are frequently mentioned in the context of SE and health include people who have experienced homelessness, people who have addiction issues, people who engage in sex work, people from the Roma and Traveller communities and others [15–17]. The 2008 WHO World Health Report advised that making primary healthcare universal would ensure that “health systems contribute to health equity, social justice and the end of exclusion” [18]. This report and a subsequent WHO Europe Regional Office report on poverty and SE reinforced the significance of the role that health systems have in addressing SE and improving the health status the population as a whole [19]. Its authors wrote that addressing the health needs of SE people should “be grounded in a human rights approach to health and the values and principles of primary health care”, and highlighted the need to include “communities experiencing poverty and social exclusion in the design, implementation, monitoring and evaluation of policy and practice” [19]. Recent research on the links between SE and health include a systematic review and meta-analysis by van Bergen et al. [12] found that exclusion was linked to adverse health outcomes, with consequences seen particularly in the area of mental health [12]. In the last decade there have been some advances with the adoption of an ‘inclusion health’ approach to the provision of care, the conduct of research and the development of policies and services for people who are considered marginalised [13, 20]. Staggering levels of morbidity and mortality have been documented in these populations, and this has led to a sense of urgency and renewed focus on trying to develop accessible and appropriate interventions to support them [20–22].

A tool to identify SE status and the measurement of its severity, could provide a broad assessment of an individual’s vulnerability and marginalisation from the ‘mainstream’ in society. It could be used over time to monitor changes in that excluded status, and it could also highlight needs they have when assessed e.g. housing, social integration etc. In that way, individual supports and services could be appropriately targeted. Information from many individuals could be aggregated to inform policy and practice developments, and consequent funding streams. However, there are challenges associated with trying to measure SE status. A 2016 United Nations report stated that “a proper assessment of exclusion requires indicators of people’s socioeconomic status – including their income, their employment situation and whether they have access to land, housing or education and health care – but it must also take into account their subjective judgements and perceptions” [2]. There are also concerns about identifying and ‘labelling’ a person or group of people with SE status. Researchers and advocates should be judicious in using any concept or terminology that could potentially worsen the stigma faced by any group that are already considered marginalised [23].

Several research teams and civil society organisations have developed measurement tools for the construct of SE that have been created and validated in a variety of ways [24–26]. Our team conducted a scoping review of these existing measurement tools in 2018: we identified 22 of these and highlighted the differences in the approaches taken in their creation [27]. With SE being a complex and broad topic, it is not surprising that there were often great variations in the domains that researchers considered relevant to SE when developing such tools [24, 27]. The majority of the tools we found were tailored for use in mental health services, potentially reflecting the consideration given to SI as an important treatment outcome in psychiatry [28, 29]. None of the tools we identified were designed for use with people from across a range of socially excluded groups; they mainly focused on one particular group e.g. people who are homeless. More recently, we conducted qualitative research with representatives of relevant stakeholder groups that resulted in the development of a definition of SE and a novel framework to aid its conceptualisation [30]. We also asked these participants to discuss and rank a list of life domains that they felt would be important to include in a SE measurement tool.

### Aim and objectives

The aim of this research was to develop a SE measurement tool for use with individuals in any healthcare setting and with people from any socially excluded group. This was informed by the extant literature, our own published work and our clinical experience of working with socially excluded groups in healthcare settings. The objectives for this research included using inductive and deductive methods to develop a draft tool, testing that tool with experts (both academic and experts by experience), modifying the draft tool in line with feedback, piloting the tool and finally assessing the internal consistency and validity of the tool.

## Methods

The measurement tool was created in two phases: the development phase and the testing phase. Phase 1 was informed by the aforementioned prioritisation exercise and the definition and framework published in our prior qualitative work [30]. This resulted in the development of a pilot SE measurement tool. Phase 2 involved taking this pilot tool to the field for engagement with participants who were presumed to be excluded, and others who were presumed not to be excluded.

### Phase 1 – Development

#### Item generation

In order to construct the draft measurement tool, a mixed inductive and deductive approach was taken to the development of items in each of the domains of interest [31]. This meant utilising our own past empirical work and that of others as a foundation, and then building on that with this current research to develop and refine a novel measurement tool. This approach of empirically gathering information, analysing it and then mapping it conceptually has been effectively utilised by other researchers when developing measurement tools [32–36]. Here, we began with the working definition of SE that we had previously published [30]:

Social exclusion is the experience of lack of opportunity, or the inability to make use of available opportunities, thereby preventing full participation in society.

We then used that framework and a list of prioritised domains to populate a model for SE measurement (Additional File 1). These domains included ‘latent’ ones such as perceptions of agency and identity along with indicators of socioeconomic status such as employment and housing status. For the socioeconomic indicators, we incorporated some questions from the 2016 Census of the Population of Ireland [37], others from research in the UK on multiple exclusion homelessness (MEH) [38] and an international tool for the classification of homelessness [39]. As previously mentioned, we had systematically examined twenty-two measurement tools that were included in our scoping review, and we identified items from each of those measurement tools and organised them under relevant domains [27]. We then used some of those items, and others we considered relevant, to populate a draft measurement tool [40, 41]. We then modified some of these items based on our past work and experience.

#### Expert review

Thirty experts based in the Republic of Ireland were approached to give feedback on this tool and 17 of them responded. Thirteen of the 17 had a PhD degree, and six of those were professors in academic institutions. They were mainly drawn from the academic disciplines of psychology and sociology as the concept of SE itself and the development of measurement tools using psychometrics are both considered important topics in these academic disciplines. Other experts who took part were working in leadership roles in relevant non-governmental organisations (NGOs) or in health service planning nationally. Two of this group of 17 also counted themselves as experts by experience (EBE) of SE. Each expert rated the importance, relevance and clarity of every item included in the draft tool. They were also invited to comment on the domains and the overall direction of the work. The draft tool was adjusted based on the feedback.

#### Pre-testing the tool

This process began with interviewing some EBE using the three-step test-interview (TSTI) technique described by Hak et al. [42]. The method was used to try to understand the cognitive steps that that participants undertook when answering the questions. The phases involved for the researcher are monitoring the responses to each question by asking the interviewee to explain their thought process out loud, asking specific questions to probe the survey responses, and finally the conduct of a discussion on the questions and the experience of taking part [43]. This was an iterative process, and changes were made to the tool and documented after each interview. As well as meeting with EBE, we also engaged with several gatekeepers who had many years of experience working with, and advocating for, socially excluded people in their services. They gave particular feedback on the language used and the signposting of some potentially challenging questions to participants during the research. Finally, we piloted the draft tool using an online survey platform. The tool was completed online by two participants who were presumed not to be socially excluded and by four EBE (presumed to be socially excluded) in individual interviews. As well as completing the questions, all these participants were asked about the acceptability of the questions, the completion time was noted, and any difficulties that arose when answering questions covering sensitive topics were documented. In total, seven EBE, two gatekeepers and two non-EBE participants were involved in this stage of feedback. The tool was modified based on this advice and prepared for piloting.

### Phase 2 – Testing

Ethical approval for the use of this tool in the field was granted by the University of Limerick Faculty of Education & Health Sciences Research Ethics Committee in June 2019 (Reference: 2019_06_12).

#### Sampling strategy

The sample consisted of two distinct groups of participants; some who were likely to have experienced SE (Group 1), and others who were not likely to have experience of being excluded (Group 2). Using comparator groups in the development of measurement tools is useful for determining the discriminant validity of measurement tools [36, 44]. Participants from Group 1 were identified as they engaged with various NGOs and services developed for people who were often presumed to be socially excluded; for example members of the Irish Traveller community, people who were homeless and people who had a history of being in prison. It is recognised that many in these groups have significant challenges in terms of their health, and consequently have very poor morbidity and mortality outcomes [13, 20, 21]. We contacted relevant services in two Irish cities, and began a process of close engagement with the gatekeepers who were interested in supporting the research [45]. Close communication with these gatekeepers was crucial in terms of being aware of potential power imbalances and the vulnerabilities of possible participants in this group. Each Group 1 participant was given a small gratuity in acknowledgement of their contribution to the research. Group 2 participants were invited to take part using two different approaches. The research team decided that being employed at a university was a good proxy measure for non-socially excluded status, and we felt that social media or email would be good ways to reach many of these potential participants. This meant that we could get Group 2 participants to complete the measurement tool using an online survey platform, and we used a staff email list at one university, and a Twitter post encouraging university staff across the Republic of Ireland to participate. A small donation was made by the research team to a local charity for every Group 2 participant who took part in the research. Target sample size was guided by the requirements of exploratory factor analysis (EFA). Guidelines for EFA vary, with Nunnally [46] estimating that 10 participants per item was ideal, whereas Comrey and Lee [47] suggested that an absolute number of between 200 and 300 participants would give a fair to good sample.

#### Data collection

Data were collected from Group 1 participants at twelve separate sites in two Irish cities between September and December 2019. Those invited to participate included:

- Irish Traveler community health workers,
- People in supported temporary homeless accommodation
- People with complex needs living in long term homeless accommodation
- People attending low-threshold, harm reduction services for people who used drugs
- People engaged with the criminal justice system who were attending training services

The inclusion criteria were that participants were over 18 years of age, that they were engaged with a relevant gatekeeper and that they had good spoken English. One researcher conducted face-to-face interviews in private with each individual participant in Group 1. We adopted this approach in order to surmount any potential literacy or numeracy issues that can be common in socially excluded groups. The researcher used an internet-enabled tablet computer to record data on a secure survey platform (Survey Monkey). All data were anonymised at the point of collection, and the consent of each participant was documented at the time of interview. Data for Group 2 participants were collected between October 2019 and March 2020. They were again recorded on a secure survey platform, but in this case participants completed the questions themselves. These participants were reached using an email list and social media messages containing brief information on the research, the inclusion criteria and a link to the survey. Again all data were anonymised at the point of collection, and consent from each participant was verified before starting.

#### Statistical analysis

Survey data was downloaded from the online survey platform to an IBM SPSS statistical package for analysis (Version 26). Categorical socioeconomic variables were summarised using counts and frequencies. The chi-square test was used to test associations between Groups 1 and 2, and categorical variables with Fisher's exact test was used for variables with low cell counts. A 5% level of significance was used for all tests. EFA was then carried out on the 28 items that explored subjective perceptions in order to simplify complex sets of data and describing correlations between included variables [48, 49]. We chose this method as we were seeking to identify latent variables or factors of theoretical relevance, and we used a common factor model called principal axis factoring [50]. This extraction method is useful when seeking an explanation for common variance in data. Direct oblimin rotation, an oblique form of factor rotation, was used as we expected our factors to be correlated [51]. The appropriateness of the data for this process was assessed by checking inter-item correlations, the determinant of the correlation matrix and by using Bartlett’s Test of Sphericity and the Kaiser–Meyer–Olkin measure of sampling adequacy (KMO) [52]. When deciding how many factors to retain we considered the Kaiser criteria of eigenvalues > 1 and carried out a visual inspection of the scree plot [53]. We also looked at the factors that explained > 5% of the variance. We also invoked our own qualitative judgement to evaluate the proposed factor structures [50]. The internal consistency of the factors in the final solution was calculated using Cronbach’s coefficient alpha [54, 55]. Factor scores were calculated and mean factor scores were compared across Group 1 and Group 2 participants using independent samples t-tests. A 5% level of significance was used for all tests.

## Results

### Phase 1

#### Item generation

Phase 1 was informed by prior qualitative work, and it resulted in the development of a pilot SE measurement tool. This draft measurement tool comprised 62 items, some of which were ‘latent’ items (28 items), and others that were considered objective indicators of socioeconomic and exclusion status (34 items).

#### Expert review

Following the expert analysis of the draft tool, a number of modifications were made (Table 1). The total number of domains included in the tool did not change, but seven items had wording changes, eight had adjustments to their Likert scales, and a further eight items had changes made to both wording and the Likert scale. One item was moved to the demographics section, and another item was removed altogether.

**Table 1:** Changes based on expert input

Changes to	Question	Change made	Rationale
Question wording	LGBTQ + status	•Wording changed from ‘Do you identify as a member of the LGBTQ + community?’ ‘Do you identify as LGBTQ + ?’	EBE had issues with the word ‘community’
	Housing	•Wording changed from ‘Where do you live?’ ‘What type of place do you live in now?’	Some answer options not covered under initial question e.g. ‘roofless’
	Housing	• ‘Couch surfing’ added as an option	Changed to reflect the current context of homelessness in Ireland
	Financial	•Wording changed from ‘How much money did you receive in to your pocket last week?’ ‘How much money did you receive from any source last week?’	Clarity of initial question wording was questioned, also ‘from any source’ was added so as to include income from activities such as sex work or asking people on the street for money
Likert scale answer options	Various	•Wording of Likert options changed •One Likert option removed •Changed to agree – disagree scale	Feedback from experts on clarity
Question wording & Likert scale options	Various	•Wording of Likert options changed •Changed to easy – difficult scale •Changes to question wording from ‘how do you feel about…’ to ‘how easy is it for you to…’	Feedback from experts on clarity
Questions moved or removed	Work status	•Moved to demographics	Feedback from experts on clarity
	How I use my time	•Question ‘how I spend my time’ removed	Feedback from experts on clarity

#### Pre-testing the tool

The tool was then evaluated by seven EBE, two gatekeepers and two participants who were presumed not to be excluded. The feedback incorporated comments on all aspects of the research from the initial information given to potential participants, to the resources and supports that should be offered to anyone who did take part in the research (Table 2).

**Table 2:** Changes based on pre-testing

Question / Element	Feedback	Change & Rationale
Consent & Participant Information Leaflet	Terms like ‘data’ and ‘explicit consent’ may intimidate or make participants nervous about taking part	No modification was made to these as they were required to be included as per ethical approval, but researcher more conscious of need to explain these terms to participants where required
Throughout	Researcher needed to show more awareness of emotional impact of questions, and signposting areas that might be difficult	Challenging questions, and cumulative ‘burden’ of asking a series of questions to each participant on potentially very difficult parts of their life
Throughout	Mention that the support of gatekeepers is available if this research has brought on any distress	New lines added at very end; ‘Thank you for taking part in this research. If the questions have upset you or brought up any issues you would like to discuss please let me know and I will speak to the gatekeeper at this service.’
LGBTQ + status	What does LGBTQ + mean? – may have to explain this	Use full term, not abbreviation, when asking the question
Challenges faced	The word ‘begged’ can be upsetting or offensive	Question changed to ‘have you ever asked people on the street for money?’
Money	Be mindful of possibility that people may be earning from non-conventional sources e.g. sex work, and may be embarrassed about that	New awareness of this when asking particular question

Following the adaptation of the draft tool based on the advice of these experts and EBE, the final draft tool was prepared and ready for piloting.

### Phase 2

Phase two involved taking this pilot tool and seeking the participation of both people who were presumed to be excluded, and others who were presumed not to be excluded. Statistical testing was carried out to try to understand the data and test the tool.

#### Participant demographics

Two hundred and seventy-six participants were recruited for this phase of the study; 127 of those were engaged with various services for vulnerable people and were therefore presumed to be socially excluded (Group 1), and 149 were university staff and so were presumed unlikely to be socially excluded (Group 2). While the age ranges were spread evenly in the two groups, the gender balance was very different with male participants being in the majority in Group 1 (Table 3). There was similar representation of people who identified as LGBTQ + in both groups (11.1% of Group 1, and 8.2% of Group 2). Regarding ethnic and cultural background, Group 2 had a profile very similar to the general population of Ireland in the 2016 Census, while Group 1 was more diverse [56].

**Table 3:** Demographics by group (*n* = 276)

Demographic variables		Group 1 – Presumed SE (*n* = 127)	Group 2 – Presumed not SE (*n* = 149)	*p*-value
Age	18–24	6 (4.7%)	6 (4.1%)	0.81
	25–34	42 (33.1%)	42 (28.6%)	
	35–44	38 (29.9%)	50 (34.0%)	
	45–54	27 (21.3%)	33 (22.4%)	
	55–64	11 (8.7%)	15 (10.2%)	
	65 +	3 (2.4%)	1 (0.7%)	
	Missing values	0	2	
Gender	Female	29 (22.8%)	118 (80.3%)	< 0.001
	Male	96 (75.6%)	29 (19.7%)	
	Other	2 (1.6%)	0 (0.0%)	
	Missing values	0	2	
Ethnic or cultural background	White Irish	98 (77.2%)	123 (83.7%)	0.005
	White Irish Traveller	12 (9.4%)	1 (0.7%)	
	Any other White background	10 (7.9%)	17 (11.6%)	
	Black African	3 (2.4%)	1 (0.7%)	
	Any other Black background	0 (0.0%)	0 (0.0%)	
	Chinese	0 (0.0%)	1 (0.7%)	
	Any other Asian background	1 (0.8%)	2 (1.4%)	
	Other, including mixed background	3 (2.4%)	2 (1.4%)	
	Missing values	0	2	
How long living in Ireland	< 5 years	6 (4.7%)	6 (4.1%)	0.56
	5–10 years	3 (2.4%)	7 (4.8%)	
	> 10 years	118 (92.9%)	134 (91.2%)	
	Missing values	0	2	
Identify as LGBTQ +	No	112 (88.9%)	135 (91.8%)	0.54
	Yes	14 (11.1%)	12 (8.2%)	
	Missing values	1	2	

Differences between the two groups were most evident in terms of work status, the amount of money the participants had received in the previous week, current accommodation and highest level of education (Table 4). We found 92.9% of the participants in Group 1 were not working, and the majority of these (44.9%) had disability status which requires a medical diagnosis of a long-term incapacitating condition. The high percentage of participants from Group 2 who were working full-time reflects the sampling strategy we utilised.

**Table 4:** Socioeconomic factors by group (*n* = 276)

Socioeconomic factor		Group 1 – Presumed SE (*n* = 127)	Group 2 – Presumed not SE (*n* = 149)	*p*-value
Work status	Not working: job seeker	49 (38.6%)	0 (0.0%)	< 0.001
	Not working: other	6 (4.7%)	0 (0.0%)	
	Not working: disability	57 (44.9%)	0 (0.0%)	
	Not working: retired	2 (1.6%)	0 (0.0%)	
	Not working: student	4 (3.1%)	10 (6.8%)	
	Working: self-employed	0 (0.0%)	3 (2.0%)	
	Working: part-time	8 (6.3%)	15 (10.2%)	
	Working: full-time	1 (0.8%)	119 (81.0%)	
	Missing values	0	2	
Current accommodation	Roofless—sleeping rough, night shelter accommodation	27 (21.3%)	0 (0.0%)	< 0.001
	Houseless—homeless, women’s shelter or immigrant accommodation	77 (60.6%)	0 (0.0%)	
	Insecure—insecure accommodation, threat of eviction or violence	1 (0.8%)	1 (0.7%)	
	Insecure—couch surfing	1 (0.8%)	1 (0.7%)	
	Inadequate—temporary structures, unfit/overcrowded housing	9 (7.1%)	0 (0.0%)	
	Good housing—rented or owned, live with family	12 (9.4%)	142 (98.6%)	
	Missing values	0	5	
Money from any source in the last week	< €250	121 (95.3%)	31 (21.4%)	< 0.001
	€250-€350	6 (4.7%)	7 (4.8%)	
	€351-€450	0 (0.0%)	7 (4.8%)	
	€451-€550	0 (0.0%)	19 (13.1%)	
	> €550	0 (0.0%)	81 (55.9%)	
	Missing values	0	4	
Highest level of education	No formal education or training	6 (4.7%)	0 (0.0%)	< 0.001
	Primary school	39 (30.7%)	0 (0.0%)	
	Lower secondary school, to Junior Certificate	29 (22.8%)	1 (0.7%)	
	Upper secondary school, to Leaving Certificate	34 (26.8%)	1 (0.7%)	
	Any qualification after secondary school, e.g., degree, diploma	19 (15.0%)	142 (98.6%)	
	Missing values	0	5	

Questions reported in Table 5 were used to document both past and recent challenges faced by the research participants. A history of imprisonment, past use of ‘hard’ drugs, having taken part in on-street drinking and having had to steal from shops in order to survive were found in the responses of more than half of Group 1 participants.

**Table 5:** Challenges faced by group (*n* = 276)

Challenges		Group 1 – Presumed SE (*n* = 127)	Group 2 – Presumed not SE (*n* = 149)	*p*-value
Have you ever…	Been in prison or young offenders institution	72 (57.6%)	0 (0%)	< 0.001
	Had a period in life when had six or more alcoholic drinks on a daily basis for more than a week	74 (59.2%)	15 (10.6%)	< 0.001
	Used 'hard' drugs e.g. heroin, cocaine, amphetamine, ecstasy, GHB	75 (60.0%)	12 (8.5%)	< 0.001
	Injected drugs	44 (35.2%)	0 (0%)	< 0.001
	Been admitted to hospital because of a mental health issue	62 (49.6%)	7 (4.7%)	< 0.001
	Been in state care as a child	35 (28.0%)	5 (3.5%)	< 0.001
	Been involved in street drinking	68 (54.4%)	17 (12.1%)	< 0.001
	Shoplifted because needed things like food, drugs, alcohol or money for somewhere to stay	71 (56.8%)	4 (2.8%)	< 0.001
	Asked people on the street for money	58 (46.4%)	1 (0.7%)	< 0.001
	Had sex or engaged in sex act in exchange for money, food, drugs or somewhere to stay	8 (6.4%)	2 (1.4%)	0.05
In the last month have you…	Used 'hard' drugs	41 (32.8%)	0 (0%)	< 0.001
	Injected drugs	18 (14.4%)	0 (0%)	< 0.001
	Been admitted to hospital because of a mental health issue	8 (6.4%)	0 (0%)	0.002
	Asked people on the street for money	23 (18.4%)	0 (0%)	< 0.001
	Had sex or engaged in sex act in exchange for money, food, drugs or somewhere to stay	0 (0%)	1 (0.7%)	1.0

#### Exploring dimensionality and items

The 28 items that explored subjective perceptions and judgements were considered suitable for EFA. The correlation matrix of items identified one item that was weakly correlated (correlation coefficient *r* < 0.3) with most of the other items, meaning it was unlikely to share common factors with them, and so it was removed. Three other items were very highly correlated with each other (*r* > 0.7), and so they were considered redundant. Two items had weak factor loadings (< 0.3) on all factors, and these were removed. The suitability of the remaining 22 items for EFA was supported by a KMO index of 0.903 and by Bartlett’s Test of Sphericity (*p* < 0.001). The final factor solution was guided by visual inspection of the scree plot, eigenvalues > 1 and interpretability (Additional File 2). This four-factor solution with rotated factor loadings for the 22 items is seen in Table 6. Factor 1 accounted for 35.30% of the variance, factor two 8.19%, factor three 6.32% and factor four accounted for 5.57%. The cumulative total variance explained by these four factors was 55.40% (Additional File 2).

**Table 6:** Pattern matrix

Pattern Matrix^a^				
	Factor			
	1	2	3	4
You are treated with less respect than others	**.747**	.044	-.071	.106
People act as if they think you are not smart	**.743**	-.027	-.038	.062
People act as if they think you are dishonest	**.724**	.031	.128	-.093
People act as if they’re better than you are	**.710**	.006	-.024	.104
You receive poorer service than other people at restaurants or shops	**.608**	.122	.060	-.080
People act as if they are afraid of you	**.585**	.073	.006	.002
You are called names or insulted	**.463**	.025	.091	.262
I feel like part of a group or community	-.085	**.720**	.095	.153
My opinions are respected when I have my say in my community	.085	**.692**	-.010	-.063
I feel safe in my community	.143	**.687**	.043	.045
I know the rules in my community, and I can fit in with them	.057	**.662**	.025	-.038
I am able to get support from family or friends when I need	.040	**.543**	.091	.228
I talk about current affairs with others	-.077	.169	**.704**	-.079
I feel free to express my beliefs (e.g. political or religious)	-.096	.145	**.698**	.026
My actions just happen without my intention	.107	.009	**.535**	.157
I feel a strong need for other people’s advice and guidance	.154	-.126	**.423**	.174
I often find it difficult to determine what I really want	.260	-.094	**.397**	-.004
I can easily manage a new problem on my own	.158	.169	**.315**	.073
How satisfied are you with your current money situation?	-.037	-.096	.097	**.715**
How satisfied are you with your work status?	.095	.108	-.088	**.587**
How would you describe your overall physical health today?	.143	.124	.006	**.516**
How would you describe your overall mental health today?	.007	.202	.133	**.476**

Extraction Method: Principal Axis Factoring

Rotation Method: Oblimin with Kaiser Normalization

^a^Rotation converged in 7 iterations

Factor 1 (Societal rejection) had factor loadings > 0.4 on seven items concerned with the individual’s perception of how they were seen by others and the way they were treated in daily life. Factor 2 (Ostracism) had factor loadings > 0.5 on five items that asked about the level of engagement the person had with family, friends and the wider community. Factor 3 (Uncertainty in engagement) had factor loadings > 0.3 on six items related to an individual’s confidence and openness when engaging with others, and when dealing with uncertainty and life-events. Factor 4 (Fundamental elements) had factor loadings > 0.4 on four items that addressed income and work status, along with health.

The inter factor relationships of these four factors are seen in Additional File 2, and the factors were found to be correlated (r ≥ 0.3). The internal consistency of the factors was high: Factor 1 had an alpha of 0.88, Factor 2 had an alpha of 0.84, Factor 3 had an alpha of 0.78 and Factor 4 had an alpha of 0.75. The reliability of all the items combined resulted in an alpha of 0.91. Table 7 details the descriptive statistics and Cronbach’s alpha for the final items and factors. We have also included the inter-item correlations as Additional File 3.

**Table 7:** – Item descriptive statistics (*n* = 276) and Cronbach’s alpha (factor and overall)

		Mean	SD	Range (Min–Max)	Missing (n)	Cronbach’s alpha
Item	**Factor 1 – Societal Rejection**					0.88
1	You are treated with less respect than other people are	2.00	1.38	0–4	18	
2	People act as if they think you are not smart	1.52	1.44	0–4	18	
3	People act as if they think you are dishonest	0.90	1.32	0–4	18	
4	People act as if they’re better than you are	1.99	1.46	0–4	18	
5	You receive poorer service than other people at restaurants or shops	0.99	1.41	0–4	18	
6	People act as if they are afraid of you	0.85	1.37	0–4	18	
7	You are called names or insulted	1.36	1.49	0–4	18	
	**Factor 2 – Ostracism**					0.84
8	I feel like part of a group or community	1.53	1.24	0–4	18	
9	My opinions are respected when I have my say in my community	1.46	0.98	0–4	18	
10	I feel safe in my community	1.27	1.11	0–4	18	
11	I know the rules in my community, and I can fit in with them	1.03	0.84	0–4	18	
12	I am able to get support from family or friends when I need it	1.28	1.23	0–4	18	
	**Factor 3 – Uncertainty in Engagement**					0.78
13	I talk about current affairs with others	1.49	1.29	0–4	21	
14	I feel free to express my beliefs (e.g. political or religious)	1.63	1.39	0–4	19	
15	My actions just happen without my intention	1.61	1.09	0–4	11	
16	I feel a strong need for other people’s advice and guidance	2.24	1.16	0–4	12	
17	I often find it difficult to determine what I really want	2.11	1.19	0–4	12	
18	I can easily manage a new problem on my own	1.53	1.04	0–4	12	
	**Factor 4 – Fundamental Elements**					0.75
19	How satisfied are you with your current money situation?	2.95	1.25	1–5	4	
20	How satisfied are you with your work status?	2.73	1.20	1–5	7	
21	How would you describe your overall physical health today?	2.53	1.17	1–5	5	
22	How would you describe your overall mental health today?	2.46	1.15	1–5	6	
	All items combined					0.91

In order to ascertain whether the factors were useful for discriminating between our two participant groups, we also compared their mean scores (Table 8). The items in our tool were designed to result in a high score if a participant answered in a way that indicated that they were socially excluded. Group 1, the group that were presumed more likely to be SE, scored higher for all four factors.

**Table 8:** Mean scores by factor and group

		Group 1 – Presumed SE	Group 2 – Presumed not SE		
	Possible score range	**Mean score (Standard deviation)**	**Mean score (Standard deviation)**	95% Confidence Interval of Difference	*P*-value
*Factor 1 – Societal Rejection*	0 – 28	**14.18 (7.64)**	**5.62 (4.50)**	6.99 to 10.13	< 0.001
*Factor 2 – Ostracism*	0 – 20	**8.98 (3.62)**	**4.46 (3.67)**	3.62 to 5.41	< 0.001
*Factor 3 – Uncertainty in Engagement*	0 – 24	**14.44 (3.67)**	**7.30 (3.06)**	6.31 to 7.98	< 0.001
*Factor 4 – Fundamental Elements*	0 – 20	**12.94 (3.04)**	**8.74 (3.87)**	3.50 to 4.91	< 0.001

## Discussion

The aim of this research was to develop a tool to measure the SE status of a person attending a healthcare setting. It is recognised that SE is an important contributory factor to poor health, and that it should be measured in terms of both the socioeconomic status and the perceptions of the individual about their treatment by society. The early stages of the research process were informed by published work and clinical experience. We then engaged with a wide variety of EBE, their advocates and academic experts in order to improve the draft tool. Finally, we conducted a pilot study with participants in the field to assess the psychometric properties of the tool.

### Statement of principal findings

The findings indicate that we have developed a measurement tool with satisfactory psychometric properties. For the socioeconomic questions, we have created a combination of items that cover a broad array of domains of relevance. For the more subjective items, using EFA we have worked out a four-factor structure that is conceptually clear and makes sense in light of the extant literature and the experience of the researchers. The internal consistency of these four factors is good, and this reassures us that the items are measuring the same underlying construct. A panel of EBE, relevant gatekeepers and senior academics extensively evaluated the content validity of both the socioeconomic and subjective items. Discriminant validity is achieved when a measurement tool is able to distinguish between participant groups that are expected to preform differently when answering, and we have shown that this tool did that effectively for people who were presumed to be excluded and others.

### Discussion of findings in relation to the existing literature

In relation to the sample of participants who we engaged for this research, one obvious difference was that the gender balance in Group 1 (presumed excluded) was heavily skewed toward males (75.6% M, 22.8% F), while it was quite the opposite for Group 2 (19.5% M, 79.2% F). This dominance of male representation has been recorded frequently in past research with marginalised groups, particularly in terms of homelessness in Ireland [57, 58]. For the gender balance of Group 2 participants, our sample is closer to the figure presented in a 2018 survey of Irish higher education institutions where 54% of staff were female [59]. Also, the finding that 21.3% of participants from Group 1 reported sleeping outdoors or staying in very precarious ‘night-only’ shelters at the time of the research reflects good participation rates from a subset of Group 1 who are often considered people who are ‘hard-to-reach’ and difficult to engage in academic research. Overall, looking at the results displayed in Table 5 for Group 1, we can see that the majority of the sample we met had faced profound difficulties in the past, leading them to resort to certain survival behaviours such as shoplifting or sex-work. We also found that almost a third of the participants from Group 1 had recently used hard drugs (32.3%), and 18.1% had recently asked people on the street for money, reflecting that the exclusion and marginalisation of these participants was likely still an issue for them at the time of the study. Our ability to engage with people who were so excluded was because of careful planning and sensitive engagement with gatekeepers and participants over a period of time [45, 60].

When we look at the EFA, the four-factor structure we have identified shows us that this tool can empirically capture a broad range of subjective experiences from participants. This has allowed us to encompass a variety of experiences from how accepted they feel by others in society, to confidence in their own ability to manage day-to-day problems, and subjective opinions on their own health. The inclusion of a broad array of component domains is also seen in some existing SI tools that were developed primarily for use with patients with mental health difficulties [32, 61]. This also speaks to the limitations of approaches that only concentrate on ‘hard’ measures such as income levels and educational status, without considering the experiences and opinions of the people affected [2]. On reviewing Table 7 and the mean scores achieved across each of the factors, it suggests that Group 1 participants have higher scores for all four factors, making them more likely to be socially excluded. It is interesting to note that the Group 1 score for Factor 2 was 8.98 out of a maximum possible score of 20. The Group 2 score for this same factor was clearly less at 4.46, but it is interesting to explore why the Group 1 score might be relatively low. The majority of items under that factor focus on community, and feeling involved and safe in a community. Some socially excluded groups have described being part of ‘micro-communities’ that offer support and non-judgement away from mainstream society [30, 62]. Other authors such as Dingle et al. [63] have found that breaking ties with these marginalised social groupings may be a necessary step in order to begin to leave exclusion. It is possible that our participants were thinking of these ‘micro-communities’ when answering the items we included under Factor 2, resulting in lower scores than the other factors.

### Strengths and weaknesses of the research

The strengths of this work include the fact that it built on a scoping review and rigorous qualitative research conducted with relevant participants [27, 30]. The research conducted for this study was methodologically robust, integrating empirical findings with the perspectives of experts (some directly affected by SE). We believe this combination makes this tool relevant and useful in seeking to capture the essence of SE. As stated, the development of trusting relationships with gatekeepers and the sensitive and low-key approach taken with possible participants who were considered excluded were key to reaching them and maintain their participation for the duration [45]. The tool addresses the gap highlighted with the new focus on inclusion health and its emphasis on looking at issues and solutions that cut across marginalised groups [20, 21]. We have now begun the development of such a measurement tool that has been tested across a range of such groups.

Although the tool has promising psychometric properties, some potential methodological limitations warrant exploration. While the number of experts involved in the Phase 1 Expert review was high, numbers involved in the Pre-testing of the tool were low and it only took place on one occasion. In terms of sample size for Phase 2, some authors have said that if there is strong data, a clear factor structure emerges and there are strong factor loadings evident, then a relatively small sample size can be justified [64]. In the development of the tool items, we involved many experts in a variety of ways in order to review the content for comprehensibility and appropriateness. Even with layers of pre-testing, there was a risk that the concept of SE would be difficult and challenging for participants from both groups to explore. To minimise this, we gave clear background information to all participants and gatekeepers prior to beginning the research and we addressed any questions that arose during the Group 1 interviews. The questions were administered to Group 1 and Group 2 participants in different ways: Group 2 participants answered an online questionnaire, while Group 1 participants had an interview where the questions were read out to them. The aim was to engage with people who may have had limited literacy and numeracy, as is common in SE groups. The finding are generalisable to other settings. We succeeded in engaging some participants who were at the extreme end of the SE spectrum. We acknowledge, however, that our Group 1 participants were attending gatekeeper services, and it is possible that the most excluded people would probably not have been working with any services. The research team was small and the resources available were finite, so we could not seek to reach other marginalised groups with known poor health status such as current prisoners or undocumented migrants. On the other hand, the engagement only with people employed in universities as Group 2 participants could be seen to be limiting. Finally, it is possible that some participants in Group 2 experienced aspects of SE despite being employed by a university. Further research with bigger samples is warranted.

### Implications for research and practice

The development of this measurement tool was comprehensive, but there is more potential research to carry out. A confirmatory factor analysis should be carried out using a new sample of participants. The sample should be tested at two specific time points to assess the test–retest reliability of the tool. The reliability and validity of the tool should be tested in other settings, and possibly in other cultural contexts and languages. Finally, a shorter version of the tool could be created, and this would be particularly advantageous for busy clinical settings and with people who often limit their engagement in those settings. This could potentially allow the creation of cut-off scores that may be useful for stratifying people into different levels of SE, allowing for more targeted actions on the provision of relevant supports and resources.

## Conclusions

This research was designed to look at SE measurement at the individual level across a number of marginalised or ‘inclusion health’ groups. In order to do this, we have combined some socioeconomic indicators with ‘latent’ measures of the more subjective elements of SE into a measurement tool. We then sought the input and advice of various EBE, people who work closely with inclusion health groups and a number academic experts in order to modify the tool. We then piloted the tool with two distinct samples and found obvious differences both in the responses to the items addressing socioeconomic issues and in the items included in the EFA. The resulting set of items can provide a robust and comprehensive tool for the measurement of SE in individuals in the community. Further evaluation and testing of this item set should be carried out in due course.

## Supplementary Information

**Additional file 1.** Framework of social exclusion.

**Additional file 2.** EFA detail.

**Additional file 3.** Inter-item correlations (*n*=276).

## Data Availability

The findings of the data analysis for this study are included in the published article and the supplementary information files. The dataset supporting the conclusions of this article is available from the corresponding author on reasonable request by a qualified applicant.

## Abbreviations

EBE: Experts by Experience
EFA: Exploratory Factor Analysis
GP: General Practitioner
HSE: Health Service Executive
KMO: Kaiser–Meyer–Olkin
LGBTQ +: Lesbian, Gay, Bisexual, Transgender and Queer/Questioning
MEH: Multiple Exclusion Homelessness
NGO: Non-Governmental Organisation
SE: Social Exclusion
SEKN: Social Exclusion Knowledge Network
SDG: Sustainable Development Goal
SI: Social Inclusion
TSTI: Three-Step Test-Interview
WHO: World Health Organization

## References

[1] Lenoir R (1974). Les Exclus: Un Francais sur Dix.

[2] United Nations (2016). Leaving No One Behind: The Imperative of Inclusive Development. Report on the World Social Situation 2016.

[3] Das MB, Evans TG, Palu T, Wilson D (2017). Social Inclusion: What does it mean for health policy and practice? - Health, Nutrition and Population Discussion Paper.

[4] Popay J, Escorel S, Hernández M, Johnston H, Mathieson J, Rispel L. Understanding and tackling social exclusion. Final report to the WHO Commission on Social Determinants of Health from the Social Exclusion Knowledge Network. World Health Organization. 2008 https://www.who.int/social_determinants/knowledge_networks/final_reports/sekn_final%20report_042008.pdf Accessed 01 Mar 2016.

[5] Piachaud D, Bennett F, Nazroo J, Popay J (2009). Report of Task Group 9: Social Inclusion and Social Mobility. Task Group Submission to the Marmot Review.

[6] Atkinson AB (1998). Poverty in Europe.

[7] Beall J (2002). Globalization and social exclusion in cities: framing the debate with lessons from Africa and Asia. Environ Urban.

[8] Gore C, Figueiredo JB (1997). Social Exclusion and Anti-Poverty Policy: A Debate.

[9] Mathieson J, Popay J, Enoch E, Escorel S, Hernandez M, Johnston H, et al. Social Exclusion Meaning, Measurement and Experience and Links to Health Inequalities: A Review of Literature. WHO Social Exclusion Knowledge Network. 2008. http://www.who.int/social_determinants/media/sekn_meaning_measurement_experience_2008.pdf.pdf Accessed 01 Feb 2016

[10] Rai SS, Peters RMH, Syurina EV, Irwanto I, Naniche D, Zweekhorst MBM (2020). Intersectionality and health-related stigma: insights from experiences of people living with stigmatized health conditions in Indonesia. Intern J Equity Health.

[11] Hankivsky O (2014). Intersectionality 101.

[12] van Bergen APL, Wolf JRLM, Badou M, de Wilde-Schutten K, Ijzelenberg W, Schreurs H (2018). The association between social exclusion or inclusion and health in EU and OECD countries: a systematic review. Eur J Pub Health.

[13] Marmot M (2018). Inclusion health: addressing the causes of the causes. The Lancet.

[14] Wilkinson R, Marmot MG (2003). Social Determinants of Health: The Solid Facts.

[15] Gill P, MacLeod U, Lester H, Hegenbarth A (2013). Improving Access to Health Care for Gypsies and Travellers, Homeless People and Sex Workers.

[16] Cabinet Office Social Exclusion Task Force & Department of Health (2010). Inclusion Health Evidence Pack.

[17] Dixon H, Povall A, Ledger A, Ashwell G (2021). Medical students’ experiences of health inequalities and inclusion health education. Clin Teach.

[18] World Health Organization (2008). The World Health Report 2008: Primary Health Care Now More Than Ever.

[19] World Health Organization (2010). Poverty, social exclusion and health systems in the WHO European Region.

[20] Aldridge RW, Story A, Hwang SW, Nordentoft M, Luchenski SA, Hartwell G (2018). Morbidity and mortality in homeless individuals, prisoners, sex workers, and individuals with substance use disorders in high-income countries: a systematic review and meta-analysis. Lancet.

[21] Luchenski S, Maguire N, Aldridge RW, Hayward A, Story A, Perri P (2018). What works in inclusion health: overview of effective interventions for marginalised and excluded populations. Lancet.

[22] Campos-Matos I, Stannard J, de Sousa E, O'Connor R, Newton JN (2019). From health for all to leaving no-one behind: public health agencies, inclusion health, and health inequalities. Lancet Publ Health.

[23] Adam C, Potvin L (2016). Understanding exclusionary mechanisms at the individual level: a theoretical proposal. Health Promot Int.

[24] Cordier R, Milbourn B, Martin R, Buchanan A, Chung D, Speyer R (2017). A systematic review evaluating the psychometric properties of measures of social inclusion. PloS one.

[25] Baumgartner JN, Burns JK (2014). Measuring social inclusion-a key outcome in global mental health. Int J Epidemiol.

[26] Coombs T, Nicholas A, Pirkis J (2013). A review of social inclusion measures. Aust N Z J Psychiatry.

[27] O’Donnell P, O’Donovan D, Elmusharaf K (2018). Measuring social exclusion in healthcare settings: a scoping review. Int J Equity Health.

[28] Sayce L (2001). Social inclusion and mental health. Psychiatr Bull.

[29] Boardman J, Davenport S, Scoping Group. Mental Health and Social Inclusion - Position Statement PS01/2009. RCPsych Publications. 2009 https://www.rcpsych.ac.uk/docs/default-source/mental-health/work-and-mental-health-library/position-statement-2009.pdf?sfvrsn=97bca29e_2 Accessed 16 Jan 2021

[30] O’Donnell P, Moran L, Geelen S, O’Donovan D, van den Muijsenbergh M, Elmusharaf K (2021). “There is people like us and there is people like them, and we are not like them.” Understating social exclusion – a qualitative study. PLoS ONE.

[31] Boateng GO, Neilands TB, Frongillo EA, Melgar-Quiñonez HR, Young SL (2018). Best practices for developing and validating scales for health, social, and behavioral research: a primer. Front Public Health.

[32] Huxley P, Evans S, Madge S, Webber M, Burchardt T, McDaid D (2012). Development of a social inclusion index to capture subjective and objective life domains (Phase II): Psychometric development study. Health Technol Assess.

[33] Huxley P, Evans S, Munroe M, Webber M, Burchardt T, Knapp M, et al. Development of a Social Inclusion Index to capture subjective and objective domains (Phase I). National Co-ordinating Centre for Research and Methodology. 2006 http://citeseerx.ist.psu.edu/viewdoc/download?doi=10.1.1.469.5761&rep=rep1&type=pdf Accessed 14 Jul 2016

[34] Korlén S, Richter A, Amer-Wåhlin I, Lindgren P, von Thiele SU (2018). The development and validation of a scale to explore staff experience of governance of economic efficiency and quality (GOV-EQ) of health care. BMC Health Serv Res.

[35] McColl MA, Carlson P, Johnston J, Minnes P, Shue K, Davies D (1998). The definition of community integration: perspectives of people with brain injuries. Brain Inj.

[36] McColl MA, Davies D, Carlson P, Johnston J, Minnes P (2001). The community integration measure: development and preliminary validation. Arch Phys Med Rehabil.

[37] Central Statistics Office (2016). Census of Population of Ireland - Sunday 24 April 2016.

[38] Fitzpatrick S, Johnsen S, Bramley G. Multiple exclusion homelessness amongst migrants in the UK. Eur J Homelessness. 2012;6(1):31-58.

[39] FEANTSA (2005). European typology on homelessness and housing exclusion.

[40] Bekker MHJ, van Assen MALM (2006). A Short Form of the Autonomy Scale: Properties of the Autonomy-Connectedness Scale (ACS–30). J Pers Assess.

[41] Tapal A, Oren E, Dar R, Eitam B (2017). The Sense of Agency Scale: A Measure of Consciously Perceived Control over One's Mind, Body, and the Immediate Environment. Front Psychol.

[42] Hak T, van der Veer K, Jansen H (2008). The Three-Step Test-Interview (TSTI): An observational instrument for pretesting self-completion questionnaires. Survey Research Methods.

[43] Hak T, van der Veer K, Ommundsen R (2006). An Application of the Three-Step Test-Interview (TSTI): A Validation Study of the Dutch and Norwegian Versions of the ‘Illegal Aliens Scale’. Int J Soc Res Methodol.

[44] Buck DS, Monteiro FM, Kneuper S, Rochon D, Clark DL, Melillo A (2005). Design and validation of the health professionals' attitudes toward the homeless inventory (HPATHI). BMC Med Educ.

[45] O’Donnell P, Elmusharaf K. Gatekeeper Engagement and the Position of the Researcher in the Development of a Social Exclusion Measurement Tool for Use in Health Care Settings. SAGE Research Methods. 2020. https://methods.sagepub.com/case/gatekeeper-engagement-position-researcher-social-exclusion-measurement-tool. Accessed 13 Jan 2021.

[46] Nunnally JC (1978). Psychometric Theory.

[47] Comrey A, Lee H, Comrey A, Lee H (1992). Interpretation and application of factor analytic results. A First Course in Factor Analysis.

[48] Kline P (1994). An easy guide to factor analysis.

[49] Pett MA, Lackey NR, Sullivan JJ (2003). Making sense of factor analysis: The use of factor analysis for instrument development in health care research.

[50] Costello A, Osborne J (2005). Best practices in exploratory factor analysis: Four recommendations for getting the most from your analysis. Pract Assess Eval.

[51] Fabrigar LR, Wegener DT, MacCallum RC, Strahan EJ (1999). Evaluating the use of exploratory factor analysis in psychological research. Psychol Methods.

[52] Tabachnick BG, Fidell LS, Ullman JB (2007). Using multivariate statistics.

[53] Ferguson E, Cox T (1993). Exploratory Factor Analysis: A Users’Guide. Int J Sel Assess.

[54] Cronbach LJ (1951). Coefficient alpha and the internal structure of tests. Psychometrika.

[55] Hinkin TR (1998). A Brief Tutorial on the Development of Measures for Use in Survey Questionnaires. Organ Res Methods.

[56] Central Statistics Office (2016). Census of Population 2016 – Profile 8 Irish Travellers, Ethnicity and Religion.

[57] O'Reilly F, Barror S, Hannigan A, Scriver S, Ruane L, Mac Farlane A (2015). Homelessness: An Unhealthy State - Health status, risk behaviours and service utilisation among homeless people in two Irish cities.

[58] O’Carroll A, O’Reilly F (2008). Health of the homeless in Dublin: has anything changed in the context of Ireland's economic boom?. Eur J Pub Health.

[59] Higher Education Authority. Higher Education Institutional Staff Profiles by Gender Dublin: HEA. 2018. https://hea.ie/assets/uploads/2018/01/Higher-Education-Institutional-Staff-Profiles-by-Gender-2018.pdf Accessed 14 Feb 2021

[60] Bonevski B, Randell M, Paul C, Chapman K, Twyman L, Bryant J (2014). Reaching the hard-to-reach: a systematic review of strategies for improving health and medical research with socially disadvantaged groups. BMC Med Res Methodol.

[61] Mezey G, White S, Thachil A, Berg R, Kallumparam S, Nasiruddin O (2012). Development and preliminary validation of a measure of social inclusion for use in people with mental health problems: The SInQUE. Int J Soc Psychiatry.

[62] Reitzes DC, Crimmins TJ, Yarbrough J, Parker J (2011). Social support and social network ties among the homeless in a downtown Atlanta park. J Community Psychol.

[63] Dingle GA, Stark C, Cruwys T, Best D (2015). Breaking good: Breaking ties with social groups may be good for recovery from substance misuse. Br J Soc Psychol.

[64] Costello AB, Osborne J (2005). Best practices in exploratory factor analysis: Four recommendations for getting the most from your analysis. Pract Assess Res Eval.

