# Supplementary material for: Developing a tool for the measurement of social exclusion in healthcare settings
Source: Int J Equity Health. 2022 Mar 15;21:35. doi: 10.1186/s12939-022-01636-1 (PMC8922776; doi:10.1186/s12939-022-01636-1)

Additional File 1 – Framework of social exclusion

Previously published in:

O'Donnell P, Moran L, Geelen S, O'Donovan D, van den Muijsenbergh M, Elmusharaf K (2021) "There is people like us and there is people like them, and we are not like them." Understating social exclusion – a qualitative study. PLoS ONE 16(6): e0253575.
https://doi.org/10.1371/journal.pone.0253575

Published in PLOS ONE under an open access license "CC-BY"

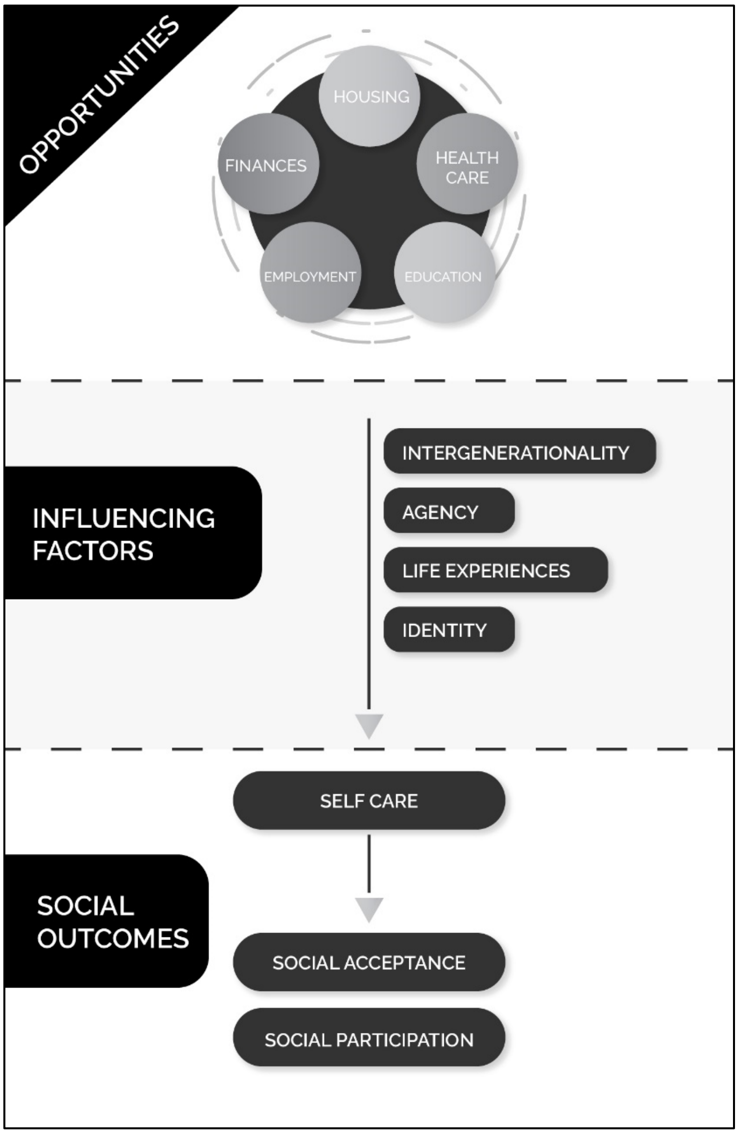

Supplement: Supplementary file 1 — Additional file 1. Framework of social exclusion. [file 12939_2022_1636_MOESM1_ESM.pdf]
