# Supplementary material for: Developing a tool for the measurement of social exclusion in healthcare settings
Source: Int J Equity Health. 2022 Mar 15;21:35. doi: 10.1186/s12939-022-01636-1 (PMC8922776; doi:10.1186/s12939-022-01636-1)
Supplement: Supplementary file 2 — Additional file 2. EFA detail. [file 12939_2022_1636_MOESM2_ESM.pdf]

Additional File 2 – EFA detail

Includes table with total variance explained, scree plot & inter-factor relationships

Extraction of initial factors

Total Variance Explained

	Initial Eigenvalues			Extraction Sums of Squared Loadings		
Factor	Total	% of Variance	Cumulative %	Total	% of Variance	Cumulative %
1	8.473	35.306	35.306	7.989	33.289	33.289
2	1.966	8.190	43.496	1.528	6.365	39.654
3	1.519	6.328	49.824	1.057	4.405	44.059
4	1.338	5.576	55.400	.830	3.458	47.517
5	1.125	4.688	60.088	.643	2.678	50.194
6	.950	3.960	64.048			
7	.862	3.593	67.641			
8	.821	3.421	71.062			
9	.755	3.146	74.208			

Scree plot

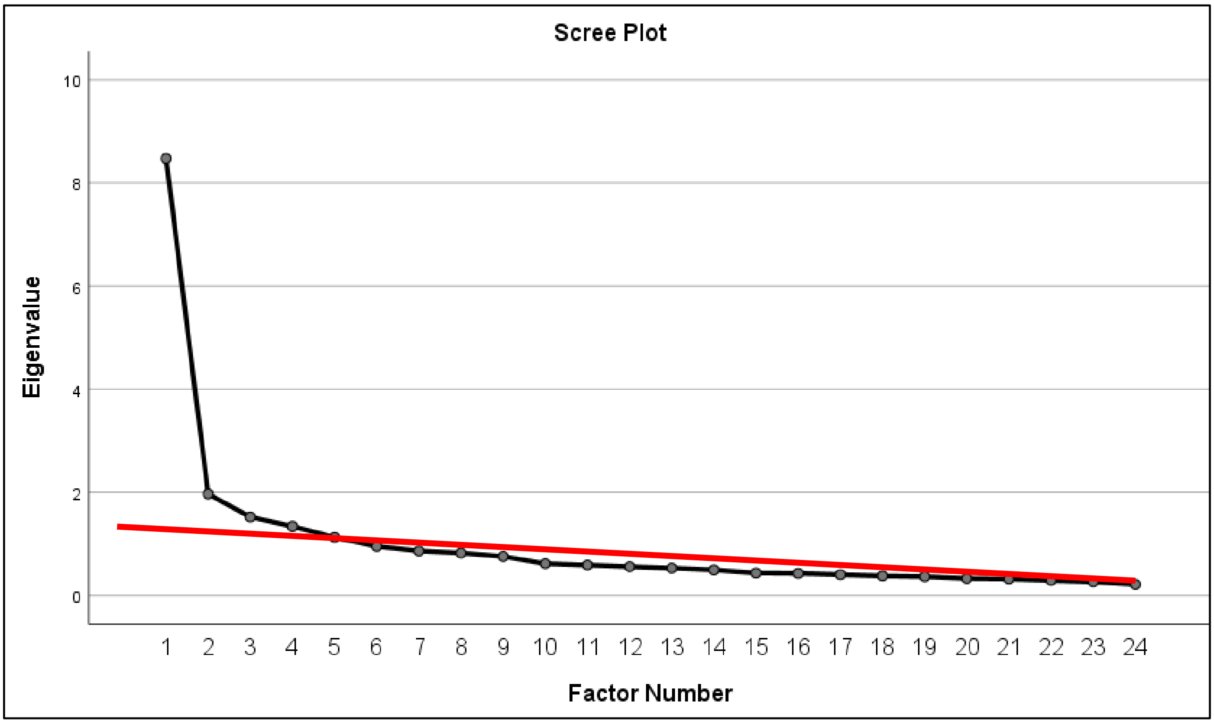

Inter-factor relationships

Factor Correlation Matrix				
Factor	1	2	3	4
1	1.000	.411	.462	.520
2	.411	1.000	.423	.333
3	.462	.423	1.000	.409
4	.520	.333	.409	1.000

Extraction Method: Principal Axis Factoring.

Rotation Method: Oblimin with Kaiser

Normalization.
