# Supplementary material for: Developing a tool for the measurement of social exclusion in healthcare settings
Source: Int J Equity Health. 2022 Mar 15;21:35. doi: 10.1186/s12939-022-01636-1 (PMC8922776; doi:10.1186/s12939-022-01636-1)
Supplement: Supplementary file 3 — Additional file 3. Inter-item correlations (n=276). [file 12939_2022_1636_MOESM3_ESM.pdf]

Additional File 3 – Inter-item correlations (n=276)

Correlations

Item

	1	2	3	4	5	6	7	8	9	10	11	12	13	14	15	16	17	18	19	20	21
2	.60																				
3	.53	.53																			
4	.65	.66	.49																		
5	.50	.48	.55	.43																	
6	.44	.38	.59	.45	.48																
7	.60	.49	.49	.52	.41	.39															
8	.29	.25	.27	.28	.32	.25	.33														
9	.31	.26	.23	.29	.27	.19	.17	.53													
10	.41	.29	.38	.33	.35	.31	.40	.63	.57												
11	.23	.23	.27	.22	.25	.26	.26	.47	.51	.58											
12	.37	.32	.39	.36	.32	.35	.37	.62	.43	.49	.45										
13	.21	.22	.26	.24	.24	.19	.28	.35	.26	.34	.27	.42									
14	.22	.20	.30	.23	.29	.22	.35	.40	.26	.30	.28	.40	.65								
15	.34	.30	.39	.35	.32	.27	.38	.32	.24	.35	.26	.35	.44	.46							
16	.31	.30	.29	.34	.15	.24	.30	.24	.13	.26	.10	.21	.26	.27	.41						
17	.31	.32	.32	.28	.27	.22	.26	.19	.22	.25	.16	.11	.25	.24	.39	.48					
18	.34	.27	.34	.29	.31	.30	.28	.33	.31	.37	.31	.32	.31	.33	.31	.42	.35				
19	.31	.28	.22	.27	.24	.24	.37	.23	.09	.19	.11	.29	.17	.23	.36	.29	.22	.25			
20	.39	.37	.24	.37	.20	.32	.36	.31	.21	.27	.22	.36	.20	.23	.20	.26	.14	.25	.51		
21	.37	.33	.38	.40	.31	.30	.43	.32	.24	.34	.23	.41	.14	.27	.40	.24	.22	.29	.38	.41	
22	.32	.29	.34	.33	.31	.23	.41	.39	.27	.39	.27	.44	.27	.30	.34	.31	.25	.34	.42	.36	.53

Shaded areas represent inter-item correlations within factors
